# How does the onset of physical disability or dementia in older adults affect economic wellbeing and co-payments for health care? the impact of gender

**DOI:** 10.1186/s12913-022-08017-y

**Published:** 2022-05-25

**Authors:** Yanan Hu, Prudence R. Carr, Danny Liew, Jonathan Broder, Emily J. Callander, John J. McNeil

**Affiliations:** School of Public Health and Preventive Medicine, Monash University, Melbourne, Australia

**Keywords:** Gender difference, Out of pocket costs, Older adults, Dementia, Physical disability

## Abstract

**Background:**

Existing studies have illustrated how the onset of physical disability or dementia negatively impacts economic wellbeing and increases out of pocket costs. However, little is known about this relationship in older individuals. Consequently, this study aimed to identify how the onset of physical disability or dementia in older adults affects economic wellbeing and out of pocket costs, and to explore the impact of gender in the context of Australia.

**Methods:**

The data was collected from a large, randomized clinical study, ASPirin in Reducing Events in the Elderly (ASPREE). Two generalized linear models (with and without interaction effects) of total out of pocket costs for those who did and did not develop physical disability or dementia were generated, with adjustment for sociodemographic characteristics at baseline.

**Results:**

We included 8,568 older Australian individuals with a mean age of 74.8 years and 53.2% being females. After adjustment for the baseline sociodemographic characteristics, the onset of physical disability did statistically significantly raise out of pocket costs (cost ratio = 1.25) and costs among females were 13.1% higher than males.

**Conclusions:**

This study highlights that classifying different types of health conditions to identify the drivers of out of pocket costs and to explore the gender differences in a long-term follow-up is of importance to examine the financial impact on the older population. These negative financial impacts and gender disparities of physical disability and dementia must be considered by policymakers.

## Background

Developments in health care over the last century have extended life expectancy [1]. This has contributed to population ageing around the world, with almost 900 million individuals currently aged 60 years or more [1, 2]. The ageing of populations has significantly increased the prevalence of physical disability and dementia in older individuals [1]. The number of older people living with dementia worldwide was over 50 million in 2020 and has been estimated to reach 82 million in 2030 [2, 3]. Also, dementia is now considered to be the primary cause of disability in older adults [2]. Similarly, the incidence of disability in people at the age of 60 years or above is 38.1% compared to 8.9% in people aged 18 to 49 years [4].

The rapid growth of disability and dementia among older adults is strongly associated with a heavy financial burden on governments, communities, families, and individuals, with the global costs of dementia estimated to reach US$2 trillion by 2030 [3]. In addition to these large societal costs, the onset of physical disability or dementia impacts the individuals' ability to participate in a healthy active life and may reduce the quality of life in a number of ways [4]. For example, the substantially increased private costs associated with accessing health care (including out of pocket costs and other indirect costs) may worsen overall economic wellbeing and lead to poverty among people living with a disability [4–8]. A prior study in Australia suggested that the poverty rate was six times higher among those with a disability than those without a disability [9]. It also has been noted that older adults with a disability face relatively higher economic costs than other age groups, with one study in Ireland estimating that costs accounted for 49.1% of household disposable income [5, 10]. Understanding out of pocket expenses is essential, as analysis of the World Health Survey data showed the lack of affordability was the leading barrier for the disabled older adults accessing health care [4]. This indicates that older individuals with a disability face double welfare implications through both age and disability [5]. As a result, great concern has been raised around the high out of pocket expenses and the affordability of health care for older adults with a disability [11–13].

While disability has a positive association with disadvantage, there are also gender inequalities compounding these disadvantages [2, 14–17]. Studies have shown that women have a greater disability or dementia at older ages than their male counterparts [2, 4, 16, 18, 19]. Older women with a disability are more likely to have lower economic resources (income and wealth), report economic hardship, and experience poverty than older men [4, 20, 21]. These gendered multidimensional disadvantage factors bring more challenges to women with disabilities at older ages [16, 19].

We therefore performed analyses using the large and high-quality ASPirin in Reducing Events in the Elderly (ASPREE) dataset, which was conducted as a part of the international clinical trial on older adults to: (i) identify the association between the onset of physical disability or dementia in older adults and individual out of pocket costs for health care and economic wellbeing; and (ii) explore the differential impact of gender.

## Methods

### Data source

The ASPREE was a double-blind, placebo-controlled randomized clinical trial of 100 mg aspirin daily on the primary endpoint of disability free survival, defined as the first occurrence of dementia, persistent physical disability, or death. ASPREE recruited 19,114 community-dwelling, healthy individuals aged 70 years or above in Australia and the U.S. (> 65 years for the U.S. minorities) between March 2010 to December 2014. Study medication for ASPREE ceased on June 12, 2017, and the median follow-up time was 4.7 years. The detailed primary results and design of the ASPREE study were reported previously [22, 23]. Overall, the ASPREE population was healthier than the general older population of Australia, due to the trial inclusion criteria. The ASPREE study was approved by the local Ethics Committees and was registered on clinicaltrials.gov (NCT01038583).

Australian participants in ASPREE were also asked to consent to a sub-study ASPREE Longitudinal Study of Older Persons (ALSOP) [24]. This involved the completion of additional medical and social questionnaires designed to collect more detailed self-reported information on medical conditions and sociodemographic characteristics, including household annual income and economic wellbeing. In addition, Australian participants were asked to consent to have their Medicare Benefits Schedule (MBS) and Pharmaceutical Benefits Scheme (PBS) claim records linked [25]. These data contain out of pocket fees paid by participants up to Sep 2016.

### Data selection and extraction

The first author and second last author designed the data selection methods, including missing data treatment. We chose a three-year follow-up period for this study as the recruitment of participants ended in December 2014, and the MBS and PBS datasets contain out of pocket information up to Sep 2016. Explanatory variables were selected based upon commonly used sociodemographic confounders.

### Measurement

#### Physical disability or dementia

The primary endpoint of this analysis was the first occurrence of physical disability or dementia during the three-year follow-up from the study entry. Participants without the documented outcome were censored at three years or at the last data they were known to be event free. Physical disability was assessed every six months by the participant’s self-reported ability to perform the 6 Katz Activities of Daily Living (ADLs), which includes walking, bathing, dressing, transferring from a bed or chair, using the toilet, and eating [26]. Physical disability was defined as participants reporting either ‘a lot of difficulty’ or ‘unable to perform’ one or more of the 6 Katz ADLs. If the Katz ADLs questions could not be administered, admission to care for assistance with activities of daily living was identified as physical disability as well. A dementia assessment was triggered if: (i) a Modified Mini-Mental State Examination (3MS) test score was lower than 77 in regular cognitive function tests [27]; (ii) a 10-point or more drop in the score (adjusted for education level attained) from baseline on the last test; or (iii) a clinical diagnosis of dementia noted in the medical records. At least six weeks after the trigger event, dementia was assessed using the Diagnostic and Statistical Manual for Mental Disorders, American Psychiatric Association (DSM-IV) criteria [28]. All components of the endpoints were adjudicated and confirmed by the respective endpoint committees blinded to treatment allocation.

#### Out of pocket fees

Out of pocket fees for all services and medications covered under the MBS and PBS were identified directly from claim records. Out of pocket fees are the amount patients paid towards the MBS and PBS subsidized medicines and health services, and the Australian Government pays the remaining costs. Out of pocket payments can be incurred for services outside of public hospitals, and for services in private hospitals, both of which are funded through the MBS; and for pharmaceuticals, which are funded through the PBS. The Federal Government will pay a proportion of the health service and pharmaceutical fees, with patients also paying an out of pocket component in some cases. Private health insurers may reimburse the patients for their out of pocket costs for services provided in a private hospital. It should be noted that out of pocket fees paid through the MBS and PBS are only part of the full range of costs paid by participants. Additional out of pocket fees can include other costs such as insurance premium costs, in-home care services, mobility aids and equipment, transportation costs, as well as home modification [6, 11, 12, 29].

In addition, out of pocket fees can vary for the same service depending on a patient’s concession status, but will not necessarily be zero as is the case in other countries. Pharmaceutical access under the PBS has a concessional rate for eligible patients (such as people with low income or seniors) and an annual PBS ‘Safety Net’, where patients pay less for medicines once they reach a threshold amount of out of pocket costs in a year. For services covered under the MBS, out of pocket charges does not have a concession rate for people with low income and are subject to capped benefits (the maximum amount of benefits payable by the Australian Government regardless of the fee charged by providers) for some health services and there is also an annual Medicare ‘Safety Net’ (patients pay less for services once they reach a threshold amount of out of pocket costs in a year) [30, 31].

For this study, total out of pocket fees were summed from Year one to Year three after the year when participants developed physical disability or dementia. For those who did not develop physical disability or dementia during the three-year follow-up, total out of pocket costs were added up over three years from the study entry. All costs were not adjusted for inflation due to the short time period.

#### Economic wellbeing

Economic wellbeing was reported based on participants’ responses to their ‘present level of income’ at the three-year follow-up ALSOP social questionnaire, with the options being: ‘very adequate’, ‘adequate’, or ‘inadequate’.

### Analytic strategy

Descriptive analysis was initially undertaken to identify the baseline demographic and socioeconomic characteristics of those who did and did not develop physical disability or dementia during the three-year follow-up. Means, standard deviations, proportions (MBS and PBS), and components (MBS) for out of pocket costs from Year one to Year three were presented, stratified by sex for those who did and did not develop physical disability or dementia. Then two generalized linear models were performed due to the skewed nature of the cost data [32]. One generalized linear model (negative binomial distribution and log link function) of total out of pocket costs from Year one to Year three for those who did and did not develop physical disability or dementia was generated, with adjustment for sociodemographic characteristics at baseline (sex, age, education attainment, annual household income, and living situation). A second generalized linear model was constructed by adding interaction effects between gender and the onset of physical disability or dementia. Finally, economic wellbeing at three-year follow-up by sex for those with and without physical disability or dementia was reported.

**Fig. 1:**
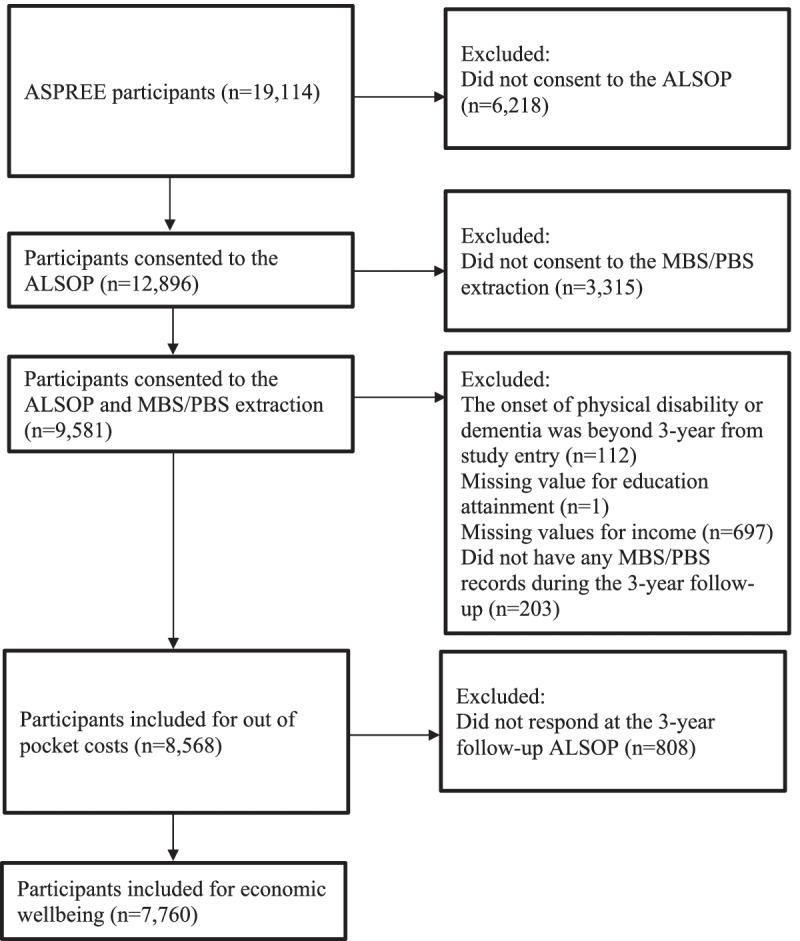
A flow diagram of sample size

A flow diagram of included participants was constructed (Fig. 1). All the statistical significance was tested using Wald Chi-Square due to the categorical nature of the data [33, 34]. All analyses were performed using SAS V9.4.

## Results

Table 1 shows the baseline demographic and socioeconomic characteristics of participants. The overall dataset consisted of 8,568 older Australian participants with a mean age of 74.8 years and 53.2% being females. The majority of participants (8,462 (98.8%)) did not develop any physical disability or dementia, while only 32 (0.4%) participants developed dementia and 74 (0.9%) individuals developed physical disability within the three-year follow-up. Females accounted for a larger share of physical disability than males (59.5% vs. 40.5%), but a lower share of dementia (46.9% vs. 53.1%). Participants who developed physical disability were on average older (76.3 years) than those who developed dementia (75.7 years); and those without dementia or physical disability (74.7 years).

**Table 1:** Demographic and socioeconomic characteristics at baseline

	No physical disability or dementia, n (%)	Developed physical disability, n (%)	Developed dementia, n (%)	Total, n (%)
Total	8,462 (98.8)	74 (0.9)	32 (0.4)	8,568
Sex				
Male	3,966 (46.9)	30 (40.5)	17 (53.1)	4,013 (46.8)
Female	4,496 (53.1)	44 (59.5)	15 (46.9)	4,555 (53.2)
Age, mean (SD) (years)	74.7 (4.0)	76.3 (5.0)	75.7 (4.4)	74.8 (4.0)
Education attainment				
< Year 9	1,201 (14.2)	13 (17.6)	5 (15.6)	1,219 (14.2)
Year 9—11	2,677 (31.6)	28 (37.8)	11 (34.4)	2,716 (31.7)
Year 12	897 (10.6)	10 (13.5)	4 (12.5)	911 (10.6)
Higher education	3,687 (43.6)	23 (31.1)	12 (37.5)	3,722 (43.4)
Annual household income				
< AU$20,000	1,175 (13.9)	18 (24.3)	7 (21.9)	1,200 (14.0)
AU$20,000—AU$49,999	4,535 (53.6)	36 (48.6)	15 (46.9)	4,586 (53.5)
AU$50,000—AU$99,999	1,606 (19.0)	9 (12.2)	4 (12.5)	1,619 (18.9)
> AU$100,000	388 (4.6)	2 (2.7)	4 (12.5)	394 (4.6)
Declined to answer	758 (9.0)	9 (12.2)	2 (6.3)	769 (9.0)
Living situation				
At home alone	2,477 (29.3)	20 (27.0)	7 (21.9)	2,504 (29.2)
At home with family, friends, or spouse	5,966 (70.5)	53 (71.6)	25 (78.1)	6,044 (70.5)
In care^a^	19 (0.2)	1 (1.4)	-	20 (0.2)

*SD* Standard deviation

^a^Includes assisted living or residential aged care facilities

 Table 2 illustrates the total out of pocket costs added from the PBS and MBS claim records from Year one to Year three stratified by gender for those who did and did not develop physical disability or dementia. On average, total out of pocket costs for females were consistently higher than for males across three health conditions. Further, women and men both paid the highest out of pocket costs when they developed dementia (women: AU$1,285.1; men: AU$969.5). All the standard deviations of out of pocket costs were relatively high compared to the means, reflecting the wide dispersion among the participants, especially for the females with dementia.

**Table 2:** Total out of pocket costs from Year one to Year three by gender and health conditions

	Total, n	Total costs, mean (SD) ($AUS)	Costs from the MBS, mean (SD) ($AUS)	Costs from the PBS, mean (SD) ($AUS)	Proportion of costs from MBS, (%)	Proportion of costs from PBS, (%)	Difference of proportion (PBS—MBS), (%)
No physical disability or dementia							
Male	3,966	867.8 (782.9)	428.6 (522.6)	439.2 (421.3)	49.4	50.6	1.2
Female	4,496	952.8 (788.8)	442.4 (524.0)	510.4 (432.1)	46.4	53.6	7.1
Physical disability							
Male	30	956.8 (672.2)	426.1 (529.8)	530.7 (367.0)	44.5	55.5	10.9
Female	44	1,210.4 (1,453.8)	457.6 (790.2)	752.8 (948.7)	37.8	62.2	24.4
Dementia							
Male	17	969.5 (900.4)	396.8 (655.5)	572.7 (417.0)	40.9	59.1	18.1
Female	15	1,285.1 (1,369.7)	675.4 (957.3)	609.7 (492.6)	52.6	47.4	-5.1

*SD* Standard deviation

**Fig. 2:**
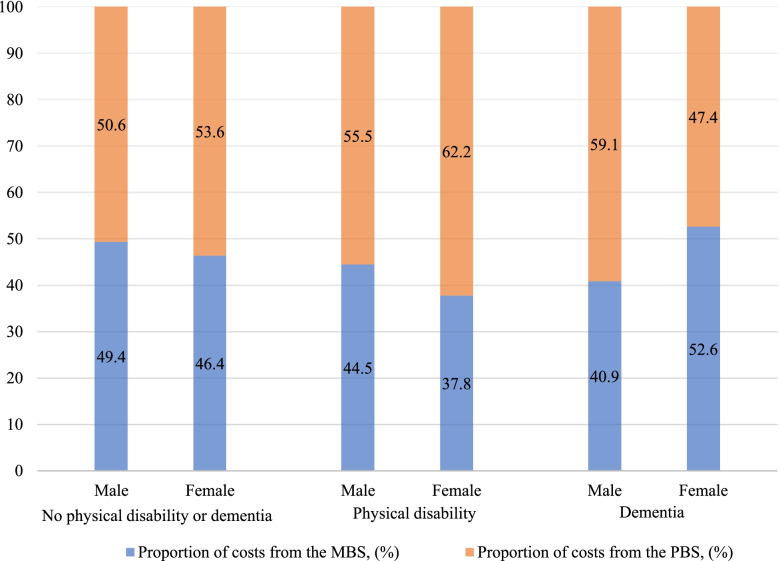
Distribution of out of pocket costs between the MBS and PBS from Year one to Year three

Table 2 and Fig. 2 show that the proportions of total out of pocket costs from the PBS claim records were consistently higher than the rates from the MBS claim records across genders and the three health states, except for females with dementia. The biggest difference was observed for females who developed physical disability where the PBS’s proportion was 24.4% higher than the share of costs from the MBS. Further, people with physical disability or dementia spent a higher share of out of pocket costs on PBS items, compared with people without disability or dementia, except for females with dementia. Females with physical disability spent most of their out of pocket costs on items from the PBS (62.2%), whereas females with dementia spent the least (47.4%) (Fig. 2).

**Fig. 3:**
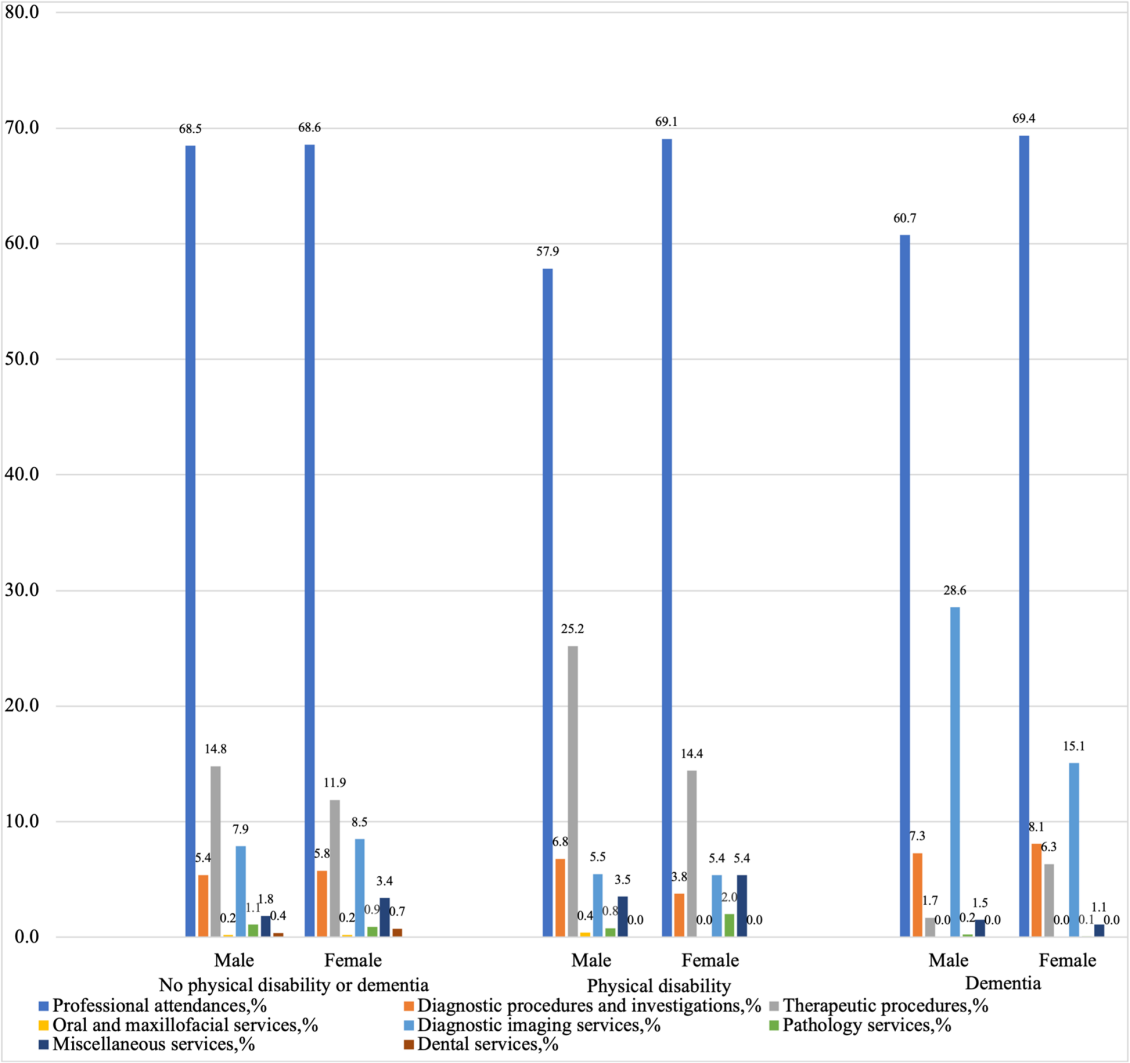
Components of total MBS costs from Year one to Year three by gender and health conditions

Table 3 and Fig. 3 further depict out of pocket costs by eight types of services accessed through the MBS. Overall, out of pocket fees from professional attendances accounted for the largest share (68.9%) and were consistently higher for females, followed by the therapeutic procedures (13.3%). The smallest share of expenditure was oral and maxillofacial services (0.2%), where older adults with dementia and females with physical disability had zero spending in this category. Moreover, the mean component of costs from dental services was zero for participants with physical disability or dementia. The second largest spending was diagnostic imaging services for older adults who reported dementia; and therapeutic procedures for the others, and both were higher in males.

**Table 3:** Components of total MBS costs from Year one to Year three by gender and health conditions

	Total costs from the MBS, mean ($AUS)	Professional attendances, mean (%) ($AUS)	Diagnostic procedures and investigations, mean (%) ($AUS)	Therapeutic procedures, mean (%) ($AUS)	Oral and maxillofacial services, mean (%) ($AUS)	Diagnostic imaging services, mean (%) ($AUS)	Pathology services, mean (%) ($AUS)	Miscellaneous services, mean (%) ($AUS)	Dental services, mean (%) ($AUS)
Total	433.8	298.9 (68.9)	24.4 (5.6)	57.7 (13.3)	0.9 (0.2)	35.9 (8.3)	4.3 (1.0)	11.7 (2.7)	2.5 (0.6)
No physical disability or dementia									
Male	428.6	293.5 (68.5)	23.1 (5.4)	63.4 (14.8)	0.8 (0.2)	33.7 (7.9)	4.7 (1.1)	7.8 (1.8)	1.6 (0.4)
Female	442.4	303.4 (68.6)	25.5 (5.8)	52.6 (11.9)	0.9 (0.2)	37.6 (8.5)	4.0 (0.9)	15.0 (3.4)	3.3 (0.7)
Physical disability									
Male	426.1	246.5 (57.9)	28.9 (6.8)	107.4 (25.2)	1.7 (0.4)	23.3 (5.5)	3.3 (0.8)	15.0 (3.5)	0
Female	457.6	316.1 (69.1)	17.2 (3.8)	65.9 (14.4)	0	24.6 (5.4)	9.1 (2.0)	24.6 (5.4)	0
Dementia									
Male	396.8	241.0 (60.7)	28.9 (7.3)	6.7 (1.7)	0	113.3 (28.6)	0.9 (0.2)	6.0 (1.5)	0
Female	675.4	468.4 (69.4)	54.5 (8.1)	42.8 (6.3)	0	101.8 (15.1)	0.4 (0.1)	7.5 (1.1)	0

Table 4 displays the cost ratios for the total out of pocket costs from Year one to Year three, adjusting for gender, education attainment, household annual income, and living situation. There was a statistically significant difference (cost ratio = 1.25, 95% CI:1.05—1.49) in total out of pocket costs for individuals who developed physical disability after adjusting for sociodemographic characteristics at baseline. Interestingly, the onset of dementia did not significantly affect the total out of pocket costs. There was also a significant discrepancy between genders; females approximately spent 1.13 times (95% CI: 1.09—1.17) more than males after accounting for baseline characteristics. Furthermore, those who were older, more educated, and earned higher annual income incurred greater total out of pocket costs.

**Table 4:** Generalized linear model of total out of pocket costs from Year one to Year three

	Cost ratio^a^ (95% CI)	Point estimate	SE	*p*-value
Intercept	-	5.9115	0.1620	< .0001
No physical disability or dementia	REFERENCE			
Physical disability	1.2517 (1.0481—1.4948)	0.4029	0.2057	0.0132
Dementia	1.0872 (0.8312—1.4221)	0.0836	0.1370	0.5415
Male	REFERENCE			
Female	1.1309 (1.0926—1.1706)	0.1230	0.0176	< .0001
Age at baseline	1.0093 (1.0051—1.0137)	0.0093	0.0022	< .0001
Education, < Year 9	0.8818 (0.8374—0.9286)	-0.1258	0.0264	< .0001
Education, Year 9—11	0.9083 (0.8731—0.9449)	-0.0962	0.0201	< .0001
Education, Year 12	0.9340 (0.8828—0.9882)	-0.0683	0.0288	0.0177
Education, Higher education	REFERENCE			
Income, < AU$20,000	0.9363 (0.8910—0.9839)	-0.0658	0.0253	0.0094
Income, AU$20,000—AU$49,999	REFERENCE			
Income, AU$50,000—AU$99,999	1.4409 (1.3773—1.5077)	0.3653	0.0231	< .0001
Income, > AU$100,000	2.2430 (2.0680—2.4327)	0.8078	0.0414	< .0001
Income, Declined to answer	1.3114 (1.2358—1.3918)	0.2711	0.0303	< .0001
Live, At home alone	1.1670 (1.1231 -1.2124)	0.1544	0.0195	< .0001
Live, At home with family, friends, or spouse	REFERENCE			
Live, In care	1.3040 (0.9276—1.8329)	0.2654	0.1738	0.1267

*CI* Confidence interval, *SE* Standard error

^a^Adjusting for sociodemographic characteristics at baseline

Table 5 further illustrates this association by estimating the main effects and interaction effects between gender and the onset of physical disability or dementia, after adjusting for confounders. However, the interaction effects between gender and the onset of physical disability or dementia were not significant, indicating that the effect of physical disability and dementia on out of pocket fees does not vary by gender.

**Table 5:** Second generalized linear model of total out of pocket costs from Year one to Year three

	Cost ratio^a^ (95% CI)	Point estimate	SE	*p*-value
Intercept	-	5.9113	0.1621	< .0001
No physical disability or dementia	REFERENCE			
Physical disability	1.2294 (0.9313—1.6229)	0.2065	0.1417	0.1450
Dementia	1.1090 (0.7674—1.6027)	0.1035	0.1879	0.5816
Male	REFERENCE			
Female	1.1309 (1.0923—1.1707)	0.1230	0.0177	< .0001
Female^*^Physical disability	1.0307 (0.7184—1.4788)	0.0302	0.1841	0.8696
Female^*^Dementia	0.9580 (0.5595—1.6403)	-0.0429	0.2744	0.8756

*CI* Confidence interval, *SE* Standard error

^a^With interaction effects and adjusting for sociodemographic characteristics at baseline

Table 6 describes the economic wellbeing of responses collected from the three-year follow-up ALSOP social questionnaire by different health conditions and genders. In total, 7,760 (90.6%) participants reported their economic wellbeing, and most of them indicated that their current level of income was ‘adequate’ (5,660, 72.9%), followed by participants who reported ‘very adequate’ income (1,556, 20.1%). Seven percent of individuals responded that their current income was ‘inadequate’. The overall pattern remains the same across gender and health conditions, with the majority reporting ‘adequate’ current income, followed by ‘very adequate’ and ‘inadequate’ income.

**Table 6:** Economic wellbeing at three-year follow-up by gender and health conditions

Economic wellbeing				
	Very adequate, n (%)	Adequate, n (%)	Inadequate, n (%)	Total, n
Total	1,556 (20.1)	5,660 (72.9)	544 (7.0)	7,760
No physical disability or dementia				
Male	715 (19.9)	2,619 (72.8)	263 (7.3)	3,597
Female	831 (20.4)	2,977 (73.0)	270 (6.6)	4,078
Physical disability				
Male	4 (15.4)	18 (69.2)	4 (15.4)	26
Female	4 (11.1)	27 (75.0)	5 (13.9)	36
Dementia				
Male	1 (7.1)	12 (85.7)	1 (7.1)	14
Female	1 (11.1)	7 (77.8)	1 (11.1)	9

## Discussion

Out of pocket costs of older adults were positively impacted by the onset of physical disability or dementia, and the effect of physical disability was significant after adjusting for socioeconomic and demographic factors at baseline. The findings from the current study are aligned with previous research that has shown having a disability, poor mental health, or poor physical function increased out of pocket costs, and suggested that the higher costs were positively associated with the prevalence of financial stress and poverty, compared to people without such health conditions [3–7, 35]. The findings from other studies also noted that such financial burden caused by out of pocket costs were associated with lower use of medical and pharmaceutical services, potentially contributing to poorer health outcomes [8, 36–41]. This demonstrates how poor health can lead to poverty and further compromises the accessibility to medical and pharmaceutical services, resulting in a cyclical relationship [8, 11, 12, 39]. These findings support concerns raised in other studies regarding the affordability of necessary medical treatments in older adults [4, 11, 12]. Further research is needed to explore the extent to which rising out of pocket fees leads to poverty among older adults with disabilities or dementia by including larger sample size and older age group.

In addition, a larger share of the total out of pocket costs was derived from prescription medication use for both genders, rather than health services such as clinician consultations or investigations. This suggests that despite government subsidization of prescription pharmaceuticals, there are still substantial co-payments from PBS-listed medications for the older population. The result is consistent with earlier studies that illustrated that older adults with chronic health conditions had considerable out of pocket spending on prescription drugs [42]. Previously, it was also found that a larger number of older Australians were experiencing affordability barriers in accessing pharmaceutical medicines [6]. Moreover, the difference in the share between charges from pharmaceutical services and medical services was more obvious for women than men. This demonstrates that older women experienced higher co-payments from Australia’s PBS than older men. Similar results were found in the context of the U.S. that older women have been shown to pay significantly higher out of pocket prescription drug costs and are more likely to bear a higher financial burden than older men [15, 40, 43, 44].

In line with our findings on out of pocket expenses across health conditions, there are also distinct gender differences in total out of pocket costs. Women paid substantially higher costs on average than men even after accounting for differences in baseline characteristics. The results are consistent with prior studies in that the financial impact of dementia was likely to be greater for women than men of the same age [45–47], and disabled women suffered from heavier costs burden and remained economically worse off than disabled men [48, 49]. Nonetheless, there is a lack of studies focusing on the gender disparities of economic consequences specifically because of the onset of physical disability or dementia. These findings imply that classifying different types of health conditions and following up a long period is essential to examine the financial impact on older adults and identify the gender differences.

### Implications for practice and/or policy

Despite the universal MBS and PBS coverage system in Australia, the onset of physical disability did significantly raise out of pocket costs after adjusting for confounders. The out of pocket cost burden fell more heavily on older women with physical disability than older men, mostly driven by prescription pharmaceutical costs. These negative financial impacts and gender disparities must be considered by policymakers. Also, the different shares and components of MBS/PBS costs have implications for policymakers in identifying the types of services on which out of pocket fees are being incurred. In order to improve these gender disparities and make health services more affordable to older adults, policymakers could lower the co-payments by removing coverage caps or providing additional support to older women with physical disability.

### Strengths and limitations

The strength of our analyses is that they drew on data from a contemporary and high-quality dataset of healthy older individuals. Nonetheless, there are a number of limitations. Although our sample size is relatively large, the number of participants who were identified with physical disability or dementia and who reported inadequate income is quite low, which may limit the generalizability of our results. The incidence numbers are also lower compared to the Australian population for physical disability (0.9% vs.8.8%) and dementia (0.4% vs. 3%) in this age group in 2016 [50, 51]. One possible reason is our participants were relatively young for people with disability and dementia as the mean age was 74.8. In addition, our sample only included three types of health conditions (without physical disability or dementia; with physical disability; and with dementia), a three-year follow-up for the occurrence of these conditions, and a three-year follow-up for out of pocket costs which is short and might not reflect the out of pocket costs as they become more disabled. As this is an ongoing study, future research could extend analyses for a longer follow-up period for both health conditions and out of pocket fees once more data is available. Finally, we were only able to include the direct out of pocket costs of health care from MBS and PBS claim records, and we were unable to quantitatively estimate how the out of pocket costs associated with economic wellbeing and poverty as a lack of income data. Indirect costs associated with the onset of physical disability or dementia also impose a large cost burden on older adults and we were unable to include them, which might underestimate the health burden. Also, the private health insurance costs and reimbursements of patients’ out of pocket fees, costs of medications not listed under the PBS were not covered, which all contributed to the amount of total expenditure.

## Conclusions

In conclusion, the onset of physical disability was associated with statistically significantly higher out of pocket costs, and costs among females were higher than males, after adjustment for baseline sociodemographic characteristics. This study provides a better understanding of how the onset of physical disability or dementia affects economic wellbeing and out of pocket expenditure in older adults by stratifying types of health conditions and differentiating by gender. It also highlights that classifying different types of health conditions to identify the drivers of out of pocket medical spending and to explore the gender differences in a long-term follow-up is of importance to examine the financial impact on the older population. Future research is recommended to include a larger sample size with the full amount of patient costs (if possible), more detailed data on current income (rather than wide income group), longer follow-up period to further explore the gender differences and financial impacts of poor health conditions on older adults.

## Data Availability

Data sharing is not applicable to this article as no new data were created or analyzed in this study.

## Abbreviations

ADLs: Activities of Daily Living
ASPREE: ASPirin in Reducing Events in the Elderly
ALSOP: ASPREE Longitudinal Study of Older Persons
CI: Confidence Interval
DSM-IV: Diagnostic and Statistical Manual for Mental Disorders, American Psychiatric Association
MBS: Medicare Benefits Schedule
3MS: Modified Mini-Mental State
PBS: Pharmaceutical Benefits Scheme
